# Serum Metabolomics of Early Postoperative Cognitive Dysfunction in Elderly Patients Using Liquid Chromatography and Q-TOF Mass Spectrometry

**DOI:** 10.1155/2020/8957541

**Published:** 2020-01-28

**Authors:** Gang Qian, YueLan Wang

**Affiliations:** ^1^Department of Anesthesiology, Shandong Provincial Qianfoshan Hospital, Shandong University, Jinan 250014, China; ^2^Department of Anesthesiology, Tongren Hospital, Shanghai Jiao Tong University, School of Medicine, Shanghai 200336, China

## Abstract

Postoperative cognitive dysfunction (POCD) is a common postoperative complication observed in elderly patients. However, the diagnosis of POCD is not very satisfactory as no specific biomarkers have been classified. It is necessary to identify new diagnostic markers to better understand the pathogenesis of POCD. We performed liquid chromatography with a time-of-flight mass spectrometer- (LC/Q-TOF-MS-) based metabolomics study to investigate POCD. A total of 40 metabolites were differentially expressed between POCD and non-POCD patients. In this study, we investigated whether phosphatidylserine (PS) (17:2/0:0), with an area under the curve value of 0.966, was a potential sensitive and specific biomarker for the diagnosis and prognosis of POCD. Pathway analysis showed that fatty acid metabolism, lipid metabolism, and carnitine metabolism were significantly altered in POCD. Network analysis indicated that nitric oxide signaling, PI3K-AKT signaling, mTOR signaling, and mitochondrial dysfunction were related to the pathogenesis of POCD. This study showed that metabolic profiling was meaningful when studying the diagnosis and pathogenesis of POCD.

## 1. Introduction

Elderly surgical patients frequently experience postoperative cognitive dysfunction (POCD), which has the symptoms of obstacles of memory, concentration, and language comprehension. These symptoms occur seven days after surgery in 25.8% of patients, and 9.9% of patients exhibit these symptoms three months after surgery; in some cases, these symptoms may be permanent. POCD often causes delayed postoperative recovery, prolonged hospital stays, and increased medical costs. POCD may have serious consequences and seriously affect the quality of life and health. Research on POCD has attracted wide attention, and significant progress has been made. While the specific mechanism of POCD is not clear, the best hypothesis regarding the pathogenesis of POCD is that it is caused by central cholinergic deficiencies, which are caused by increased regulation of inflammation by cholinergic anti-inflammatory pathways [1]. The inflammatory response to a surgical procedure was a potential factor involved in the pathogenesis of POCD. Glumac et al. found that dexamethasone could significantly reduce the inflammatory response and thereby decreased the risk of early POCD after cardiac surgery when patients were treated with dexamethasone before the surgery [2]. On the other hand, there are no sensitive markers for POCD. Therefore, it is urgent to find new biomarkers to better diagnose POCD and new therapeutic targets and drugs to treat POCD, thus reducing the increasing incidence and disease-related burden.

Some biological markers for diseases in the central nervous system (CNS) have been identified, such as genetic markers [3], RNA [4], microRNA [5], and proteins [6]. However, these biochemical entities were not specific markers for POCD. Metabonomics reflects the physiological state of the organism by analyzing the changes in metabolites in cells, tissues, organs, or organisms after genetic modification, pathological stimulation, or environmental impact [7]. Metabolites in different types of samples, including the cerebrospinal fluid, tissue, urine, and plasma, can be detected by nontargeted metabonomics analysis, which has been widely used in clinical research. At present, this method is used to identify biomarkers in central nervous system diseases [8]. To date, there have been no reports using metabolomics to discover diagnostic biomarkers in patients with POCD after general anesthesia, which suggests that it is necessary to take advantage of this novel approach to identify the potential biomarkers of POCD and to study the pathogenesis of this disease and its treatment possibilities.

In the present study, the serum of patients with POCD was analyzed with metabonomics based on LC/MS to determine the differentially expressed metabolites in the serum of early POCD patients after laparoscopic surgery with general anesthesia for gastrointestinal tumors. The aim was to explore the serological markers of early POCD diagnosis and to explore the pathogenesis of POCD.

## 2. Materials and Methods

### 2.1. Collection of Clinical Samples

Our study was a prospective study, and the purpose of our study is to screen specific metabolic markers of POCD based on a nontargeted metabolomics study. Prior to sample collection, written informed consents were obtained from all subjects. The protocols of this study were reviewed and approved by the Medical Institutional Ethics Committee of Shanghai Tongren Hospital, School of Medicine, Shanghai Jiao Tong University. Patients scheduled to undergo laparoscopic surgery for gastrointestinal tumors in Shanghai Tongren Hospital from December 2016 to December 2018 were enrolled in this study. The inclusion criteria included patients over 65 years of age, hospitalization time not less than 7 days, general anesthesia time of at least 2 hours, with the ability to read and hear to understand and cooperate with neuropsychological testing, and fluency in Chinese. The exclusion criteria included patients with hospitalization time less than 7 days, patients scheduled for carotid, intracranial, and cardiac surgery, and patients who received minimally invasive mental state examination (MMSE) scores before surgery (less than 23). Patients with diseases of the central nervous system, dementia, and psychiatric disorder, individuals currently using antidepressants or sedatives, or people with drug or alcohol dependence were excluded. We selected 20 age-matched patients as controls (non-POCD, undergoing surgery except intracranial, carotid, and cardiac surgery). After entering the operating room, patients were anesthetized with sufentanil 0.2 *μ*g/kg, propofol 1.5 mg/kg, and cisatracurine 0.3 mg/kg. During the operation, propofol 4 *μ*g/ml, remifentanil 1.5 ng/ml, and cisatracurine 0.15 mg/kg were used. If necessary, vasoactive drugs should be used to keep the fluctuation range of blood pressure within ±20% of the basic value. Fresh gas flow rate was 2 l/min, oxygen concentration was 50%, end-expiratory carbon dioxide level was maintained between 35 and 45 mmhg, nasopharynx temperature was maintained at 37°C with a warm blanket, and the maintenance range was 45-60 with a dual frequency index of an electroencephalogram. The sex ratio and average education levels were similar to those of POCD patients. The control group was given two neuropsychological tests at the same time interval (seven days), and the researchers were the same. The postoperative day tests were administered to the patients, and blood samples were obtained on the 7th postoperative day using the Montreal Cognitive Assessment (MoCA), which is a widely used screening assessment for detecting cognitive impairment. According to the MoCA scores, the inpatients were divided into two groups: POCD group: MoCA score < 24 (*n* = 20) and no-POCD group: MoCA score > 24 (*n* = 20). The detailed demographic and clinical data of the participants are presented in Table 1. The POCD and non-POCD groups did not significantly differ in age, weight, and education.

### 2.2. Chemicals and Reagents

Acetonitrile and methanol (MS grade) were purchased from Merck (Darmstadt, Germany) and Burdick and Jackson (Ulsan, Korea), respectively. Formic acid was purchased from Sigma-Aldrich Co. (St. Louis, USA). Ultrapure water was purified by a Milli-Q water purification system (Millipore, Bedford, MA). Other chemicals were of analytical grade.

### 2.3. Sample Collection

Venous blood (2 ml) was collected both before and after surgery on the same day that the neuropsychological tests were conducted. Blood samples were placed in 5 ml tubes at 25°C for 30 min, and then, the tubes were centrifuged at 3500 rpm for 10 min at 4°C. After centrifugation, the serum was labeled with the patient information and immediately stored at -80°C.

### 2.4. Sample Processing

For LC/MS analysis, 100 *μ*l of the serum was transferred to a 1.5 ml centrifuge tube, 0.3 ml of methanol with an internal standard of 10 *μ*l 2-chlorophenylalanine (4 *μ*g/ml) was added to each sample, and the mixture was vortexed for 2 min. The samples were subsequently centrifuged at 13,000 r/min for 10 min at 4°C, and 0.2 ml of the supernatant was separated from the sample.

### 2.5. LC-Q-TOF/MS Analysis

The LC (Agilent 1290) was equipped with Q-TOF/MS (Agilent 6538) (Agilent, CA, USA), which was developed for the determination of serum metabolites. An ACQUITY UPLC HSS T3 C18 column (2.5 *μ*m, 2.1 mm × 100 mm) was used for separation of all samples, and the column temperature was set at 35°C. The flow rate was 0.35 ml/min, and the mobile phases were 0.1% acetic acid in water (A) and acetonitrile (B). The compound separation was carried out under the following gradient program (time, %B): 0-2 min, 5%B; 2-13, 5-95%B; and 13-15 min, 95%B, and the posttime was 5 min. The sample injection volume was 3 *μ*l.

Mass detection was operated in both positive and negative ion modes with the following setting: drying gas (N_2_) flow rate, 11 l/min; gas temperature, 350°C; pressure of the nebulizer gas, 45 psig; Vcap, 4000 V (-3000 V); fragmentor, 120 V; skimmer, 60 V; and scan range, *m*/*z* 80-1000. All analyses were acquired using the instrument mass spray to ensure accuracy and reproducibility. Leucine enkephalin was used as the instrument reference mass (*m*/*z* 121.0509 and 922.0098 in ESI+ and 119.0363 and 966.0007 in ESI-) at a concentration of 100 ng/ml for all analyses. The MS2 analysis in the targeted MS/MS mode with three collision energies, i.e., 10, 20, and 40 eV, was used to confirm the structure of potential biomarkers by evaluating the fragment ions with the reference compound in our lab. Randomization of sample runs and the pooled quality control (QC) sample were analyzed in every six samples during the whole analysis. All the data were acquired by MassHunter software.

### 2.6. Data Processing and Identification of Metabolites

Raw data files from Q-TOF/MS were converted into the mzXML format using Agilent MassHunter Qualitative Analysis B.07.00 software (Agilent Technologies, USA), and the peak finding and chromatogram alignment were assessed with the XCMS package based on the R software package. For multivariate data analysis, we used SIMCA-P software (version 13.0; Umetrics AB, Umea, Sweden). PCA (principal component analysis), an unbiased statistical method to observe the different metabolomes between the analyzed samples, was used for the score plots of the first two principal components with each point representing one sample. A supervised analysis method, partial least-squares discriminant analysis (PLS-DA), was used for model discrimination and biomarker screening. A random permutation test was used to evaluate the overfitting of the PLS-DA model. Meanwhile, R2 (model interpretation rate) and Q2 (the model predictive ability) were used to indicate the quality of the model. The variables with VIP > 1 (variable importance in the projection) were potential differential metabolites. Furthermore, a *t*-test was used to determine if the differential metabolites selected from PLS-DA modeling were statistically significant (*p* < 0.05). To identify potential markers, we compared the precise molecular weight and fragment ion information with the databases HMDB (http://www.hmdb.ca/) and METLIN (https://metlin.scripps.edu/index.php). Differential metabolite pathway analysis and interaction network analysis were completed with IPA software (IPA, Ingenuity® Systems, https://www.ingenuity.com). MetaboAnalyst 3.0 (https://www.metaboanalyst.ca/) was used to assess the specificity and sensitivity of potential biomarkers with receptor operating characteristic (ROC) analysis.

## 3. Results

### 3.1. Patient Information

During the inclusion period, a total of 160 participants were enrolled and were asked to participate. Of these 160 participants, 60 were excluded: 30 refused informed consent, 18 had information missing, and 12 had their surgery canceled. On the 7th postoperative day, 20 of the 100 participants developed POCD. The general characteristics and intraoperative conditions were not significantly different between patients with and without POCD as shown in Table 1. At last, 20 patients developed POCD after laparoscopic surgery with general anesthesia for gastrointestinal tumors, and other 20 patients did not develop POCD after any surgery longer than 2 hours except for intracranial, carotid, and cardiac surgery. There were no significant differences in age, gender ratio, and education levels between the non-POCD group and the POCD group. There was a significant difference in the MoCA score between the two patient groups.

### 3.2. Total Ion Chromatogram

**Figure 1:**
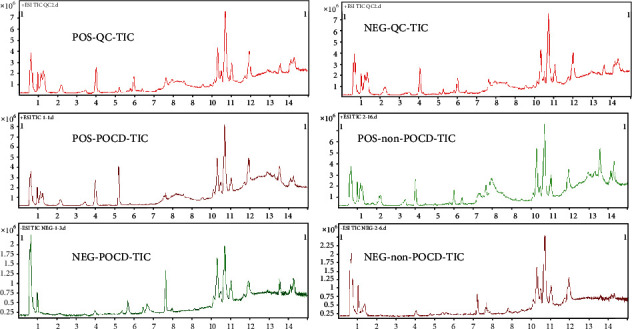
The total ion chromatogram of the non-POCD and POCD groups (+ESI, -ESI).

The representative total ion current (both positive and negative) data obtained from the serum of the QC group, POCD group, and non-POCD group are shown in Figure 1. The retention time of each major chromatographic peak in different groups with good overlapping (Figure 1) demonstrated excellent stability and reproducibility of the LCMS system during the whole sequence.

### 3.3. Multivariate Statistical Analysis

**Figure 2:**
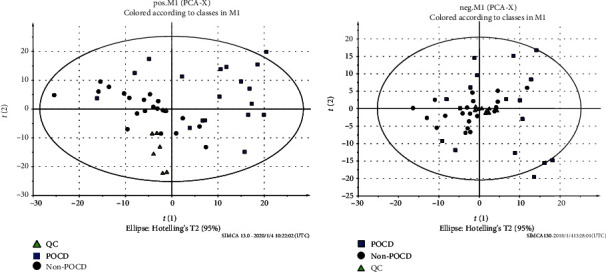
PCA score plot: (■) POCD group and (●) non-POCD group.

**Figure 3:**
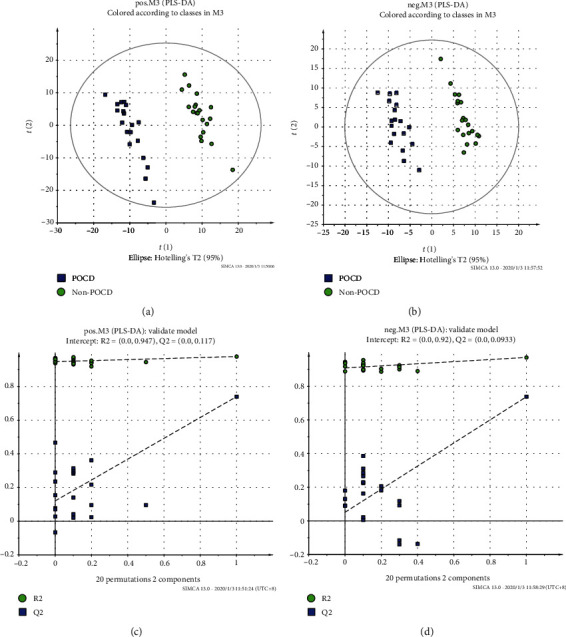
Partial least squares-discriminant analysis (PLS-DA) score plot and permutation test for the model discriminating serum samples from POCD patients and healthy controls. (a) POS-PLS-DA score plot. (b) NEG-PLS-DA score plot. (c) POS-permutation test for the model of PLS-DA. (d) NEG-permutation test for the model of PLS-DA: (■) POCD group and (●) non-POCD group.

The PCA score plot of the POCD, non-POCD, and QC groups is shown in Figure 2. There was a trend of intragroup aggregation and intergroup separation (Figure 2). The high degree of aggregation of the QC group demonstrated high stability of the LCMS system during the whole sequence. The PLS-DA was used to screen the differentially expressed metabolites between the POCD and non-POCD groups. The scores of the PLS-DA plot (Figure 3) showed a clear separation between the POCD group and the non-POCD group.

### 3.4. Differentially Expressed Metabolites

**Figure 4:**
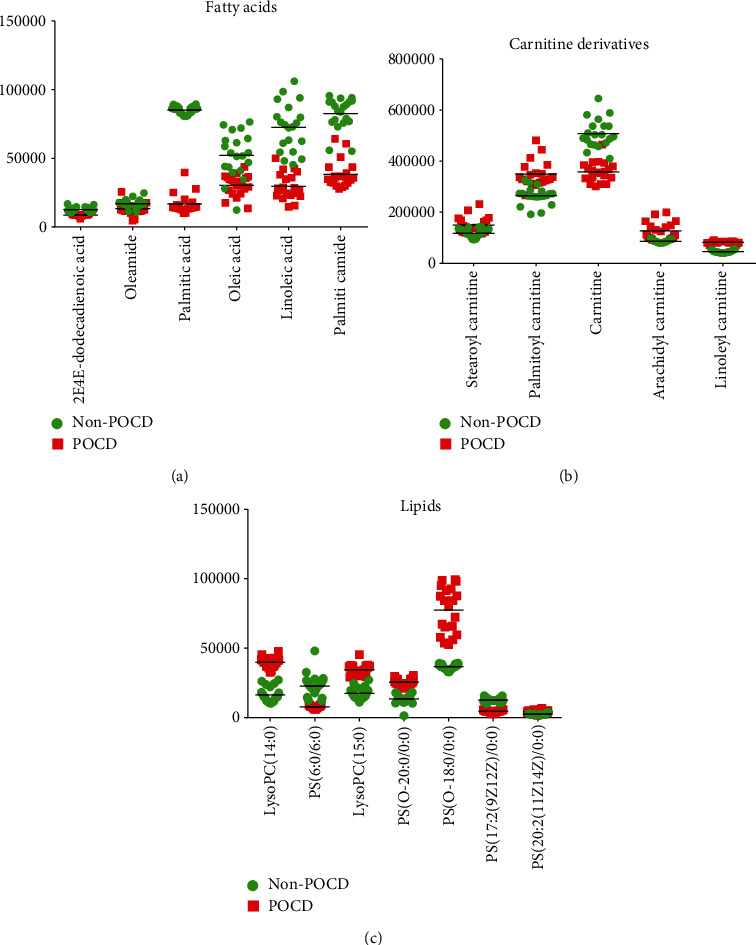
Expression levels of the significantly changed metabolites: (a) fatty acid metabolism; (b) carnitine metabolism; (c) lipid metabolism.

**Figure 5:**
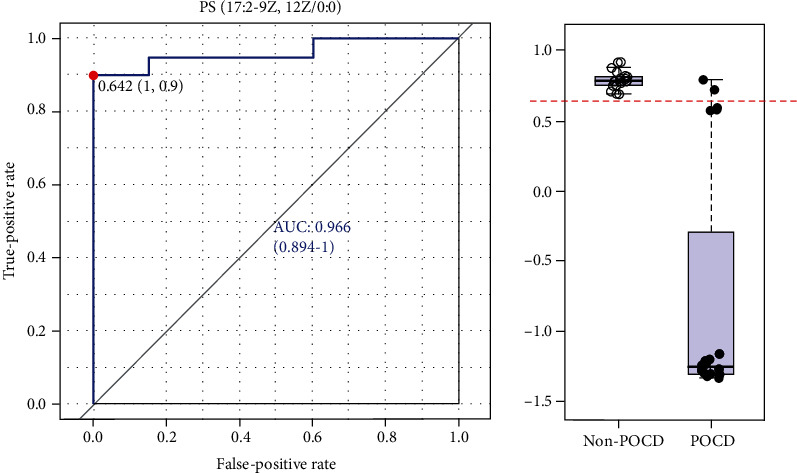
ROC curve analysis of potential serum biomarker levels for differentiating the POCD group from the non-POCD group.

**Figure 6:**
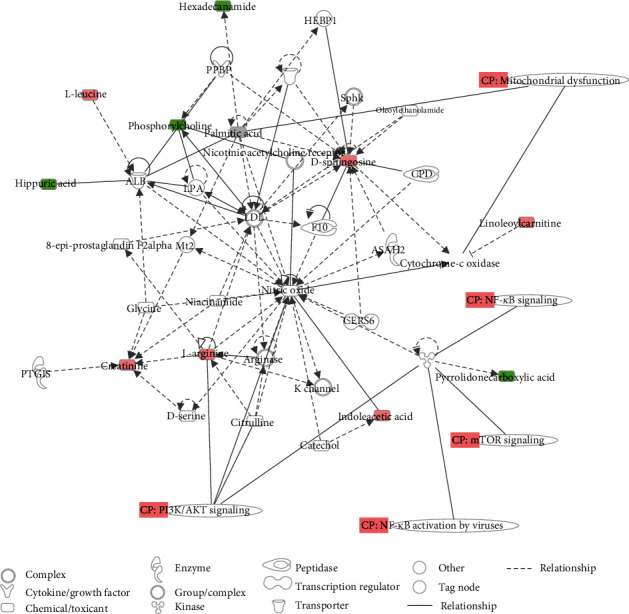
Biological network, canonical pathways, and functions related to the identified metabolites. In the network, molecules are represented as nodes, and the biological relationship between two nodes is represented as a line. Red symbols represent upregulated metabolites and green symbols represent downregulated metabolites, while blue symbols represent canonical pathways that are related to the identified specific metabolites. Solid lines between molecules show a direct physical relationship between molecules, while dotted lines show indirect functional relationships.

In our study, a variable with VIP > 1 and *p* < 0.05 was selected as a potential marker. Then, 40 differentially expressed metabolites were identified in the serum of the POCD group compared to the non-POCD group, as shown in Table 2. As a result, these metabolites were mainly fatty acids, lipids, carnitine derivatives, and other substances, as shown in Figure 4. ROC curve analysis indicated that PS(17:2/0:0), with an AUC value of 0.966, was considered a potential diagnostic marker for POCD (Figure 5). The interaction network of differentially expressed metabolites between the POCD and non-POCD groups was built based on the database of IPA (as shown in Figure 6). The five top canonical pathways included nitric oxide signaling, PI3K-AKT signaling, mTOR signaling, mitochondrial dysfunction, and nuclear transcription factor signaling pathway (NF-*κ*B).

## 4. Discussion

The incidence of POCD is high, especially in elderly patients after surgery. However, the specific mechanism of POCD is still unclear. The focus of this study was to identify serum metabolites associated with POCD and to explore the mechanisms of pathogenesis and development of POCD. In our study, 40 metabolites were found to be differentially expressed in POCD patients. Among them, fatty acids, lipids, and the carnitine derivatives were the major changing substances, as shown in Figure 4. In addition, phosphatidylserine (PS) (17:2/0:0), a kind of phospholipid, with an AUC value of 0.966, was a potential diagnostic biomarker for POCD.

According to the references, PS is the main phospholipid, accounting for 13%-15% of phospholipids in the human cerebral cortex [9]. In the plasma membrane, PS can activate key signaling pathways, such as the Akt and Raf-1 signaling pathways, which stimulate neuronal survival, process growth, and synaptogenesis [10]. PS also regulates AMPA glutamate receptors [11]. In addition, abnormal levels of PS in the synaptosomal membrane were observed in mild cognitive impairment and Alzheimer's disease [12]. In mammalian tissues, PS is synthesized by a Ca^2+^-dependent reaction of phosphatidylcholine (PC) or phosphatidylethanolamine (PE), in which the head group of the substrate phospholipid is replaced by serine [13]. By transferring phosphocholine or ethanolamine phosphate from the corresponding cytidine diphosphate derivatives to 1,2-diacylglycerol, the substrates PC and PE for PS production can be synthesized in microsomes [11]. During neuronal differentiation, PC synthesis was upregulated [14]. PC can also be synthesized in a phosphatidylethanolamine N-methyltransferase (PEMT) reaction through the three-order methylation of the ethanolamine head group of PE [15]. In the PI3K/Akt signaling pathway, Akt is the key protein for cell survival and proliferation. When PIP3 production is limited, the role of PS in promoting Akt signaling is particularly important for maintaining neuronal survival. PS also affects the properties of neuronal nitric oxide synthase, which is a key enzyme in the oxidative function of the brain. DHA contained in PC and PE is the best substrate for PS biosynthesis [16, 17]. Consequently, the PS level is high in the brain where DHA is abundant. In contrast, depletion of DHA in the brain reduces the PS level [18]. For example, dietary consumption of n-3 fatty acids can reduce DHA in the brain and increase the n-6 equivalent docosahexaenoic acid (22:5n-6, DPA-6). The results of a neuronal culture showed that the increase in PS mediated by DHA may promote the development of neurons and prevent apoptosis [19]. The discovery of DHA increasing the length and branching of cultured hippocampal neurons showed that DHA promoted the development of neurons [20], and it promotes neurogenic differentiation of neural stem cells [20]. In this study, PS(17:2(9Z,12Z)/0:0) and PS (6:0/6:0) were decreased in POCD patients, while LysoPC(14:0) and LysoPC(15:0) were increased in POCD. The disorder of LysoPCs led to energy metabolism alterations, and these were related to interruption of the cell membrane and the occurrence of inflammation with POCD. These results indicated that inhibition of neuronal development and neurogenic differentiation may be the result of the absence of PS, and POCD patients should be given supplements of foods rich in these lipids or drugs that stimulate the synthesis of PS to improve the growth of nerves to prevent POCD after surgery.

Polyunsaturated fatty acids (PUFAs) play a key role in the structure and function of the developing nervous system. Long-chain polyunsaturated fatty acids (LCPUFAs) accumulate rapidly during the development of gray matter of the brain. Lipids account for 50-60% of the adult brainstem weight, of which approximately 35% is LCPUFAs, mainly arachidonic acid (20:4n-6; AA) and DHA (22:6n-3). These LCPUFAs are obtained by biosynthesis from their respective dietary essential fatty acid precursors, linoleic acid and alpha-linolenic acid, or they can be obtained directly from dietary sources, such as eggs, fish, and meat, or, more recently, from single-cell oils [21]. LCPUFAs can change the physical properties of membranes and affect various membrane functions, including ion channels and transport, endocytosis and efflux, and the activity of membrane-binding proteins. Fatty acids are an important part of the cell membrane, and they are the largest energy reserve in the human body. However, many studies have confirmed that long-chain fatty acids can activate peripheral cells in inflammation and the innate immune response [22]. In our study, the fatty acids 2E,4E-dodecadienoic acid, linoleic acid, palmitic acid, palmitic amide, and oleic acid were decreased in POCD (as shown in Figures 4 and 5), which can be linked to energy requirements, and they particularly participate in the body's response to inflammation. These results indicated that POCD patients should eat foods, such as eggs, fish, and meat, for their essential fatty acid (EFA) precursors to assist with recovery.

L-Carnitine is an endogenous molecule and an important contributor to cellular energy metabolism. It is ubiquitous in organisms, mainly in the most metabolically active tissues, such as the myocardium and skeletal muscle. L-Carnitine transported long-chain fatty acids through the inner mitochondrial membrane to their oxidation sites to produce ATP-like energy [23]. One of the most important consequences of carnitine deficiency is the change in metabolic pathways leading to energy production [24]. Because of the importance of energy production, mitochondria have been found to be associated with aging and age-related diseases. L-Carnitine is essential for the oxidation of long-chain fatty acids in the mitochondria [25], and it is the main source of energy during exercise [26]. Increased L-carnitine content may increase the rate of fatty acid oxidation, decrease glucose utilization, preserve muscle glycogen content, and ensure the maximum rate of ATP production [27]. Acquisition of L-carnitine is not a limiting step in oxidation under normal nutritional conditions in healthy people; however, supplementation with L-carnitine can provide beneficial results in centenarians. In fact, L-carnitine can improve total muscle mass, reduce total fat mass, increase body weight, and improve walking ability [28]. L-Carnitine therapy can help relieve fatigue, depression, and muscle weakness in the elderly [29]. When administered orally, L-carnitine improves the efficiency of high-intensity muscle training. In brain tissue, L-carnitine shuttle mediates the translocation of acetyl groups from the mitochondria to the cytoplasm, thus facilitating the synthesis of acetylcholine and acetylcarnitine [30]. Neurobiological effects of acetylcarnitine include regulation of brain energy and phospholipid metabolism, cell macromolecules, synaptic morphology, and synaptic transmission of various neurotransmitters [31]. In this study, carnitine was decreased in POCD, while linoleyl carnitine, palmitoylcarnitine, and stearoylcarnitine were increased in POCD patients.

The network analysis showed that the revealed signaling pathways of POCD were selected by the IPA. These pathways included nitric oxide signaling, PI3K-AKT signaling, mTOR signaling, and mitochondrial dysfunction. These pathways are believed to be involved in the occurrence and development of neurodegenerative diseases. The PI3K-AKT signaling pathway mediates inflammation, oxidative stress, and neuronal apoptosis induced by isoflurane anesthesia or surgery, leading to cognitive impairment [32]. The activation of mTOR signal transduction can increase the production of beta-amyloid proteins and the phosphorylation of tau proteins, leading to the decline in postoperative cognitive function [33].

Although this study identified the metabolic profile of POCD and predicted their possible role in POCD, there are still some limitations. First of all, we only carried out 20-case sample size in our study, and there may be some limitations in statistical analysis. A larger sample size can achieve better statistical results. Secondly, further verified and functional studies should be done after the nontargeted metabolomics, and the possible mechanism of POCD should be further clarified.

## 5. Conclusion

A serum metabonomics method based on UPLC/MS was established to study the metabolic changes in POCD patients. The metabolic pathways perturbed in POCD have been reported. Forty differential metabolites were identified, and PS(17:0/0:0) was the potential biomarker for a POCD diagnosis. The interaction network analysis of these differently expressed metabolites in POCD was established by IPA. The results suggested that activations of the nitric oxide signaling pathway, the PI3K-AKT signaling pathway, the mTOR signaling pathway, and mitochondrial dysfunction were responsible for the pathogenesis of POCD. Our findings provide a promising approach for the study of POCD-related metabolite profiles and may provide insights into the pathogenesis of POCD.

## Figures and Tables

**Table 1:** Characteristics of non-POCD and POCD patients.

Parameter	Non-POCD	POCD	*p*
Number (*n*)	20	20	0.4
Age (months)	66.1 ± 6.50	67.5 ± 8.64	0.3
Weight (kg)	65 ± 7.1	64 ± 6.5	0.1
Gender (male/female)	10/10	10/10	0.2
Education (year)	13.0 ± 2.1	13.3 ± 1.5	0.4
MOCA	26.6 ± 2.4	13.7 ± 1.8	0.001

**Table 2:** The potential biomarkers of POCD detected by UPLC-Q-TOF/MS and their variation tendency. POCD/non-POCD: POCD group compared to non-POCD group; FC(P/C): fold change of POCD/non-POCD; ↑: upregulated; ↓: downregulated.

RT	Mass	Name	VIP	*p*	FC(P/C)	Trend
9.21	250.1205	Ubiquinone-1	2.76	1.23*E* − 05	1.95	↑
11.44	427.366	Stearoylcarnitine	1.56	2.37*E* − 02	1.20	↑
14.68	283.2875	Stearamide	2.14	1.30*E* − 03	1.98	↑
12.69	299.282	Sphingosine	1.72	1.19*E* − 02	2.13	↑
0.71	129.0427	Pyroglutamic acid	2.20	9.19*E* − 04	0.47	↓
8.95	539.3584	PS(O-20:0/0:0)	1.52	2.73*E* − 02	1.71	↑
8.12	511.3273	PS(O-18:0/0:0)	1.52	2.71*E* − 02	1.39	↑
8.99	507.2461	PS(17:2(9Z,12Z)/0:0)	3.67	1.21*E* − 11	0.10	↓
8.12	978.7156	PI(22:0/22:0)	1.80	8.06*E* − 03	1.84	↑
10.72	183.0659	Phosphocholine	2.26	6.29*E* − 04	0.50	↓
10.63	399.3349	Palmitoylcarnitine	2.39	2.51*E* − 04	1.26	↑
14.63	255.2561	Palmitic amide	1.53	2.69*E* − 02	0.41	↓
0.15	256.2406	Palmitic acid	1.40	4.39*E* − 02	0.67	↓
5.21	452.3357	PA(O-20:0/0:0)	1.48	3.19*E* − 02	1.27	↑
6.18	396.2358	PA(15:0/0:0)	1.49	3.05*E* − 02	1.70	↑
13.51	281.2716	Oleamide	1.55	2.45*E* − 02	1.97	↑
14.29	383.3398	N-Stearoyl valine	1.51	2.87*E* − 02	1.20	↑
9.94	481.3168	LysoPC(15:0)	1.47	3.33*E* − 02	1.37	↑
9.60	467.3011	LysoPC(14:0)	1.47	3.30*E* − 02	1.48	↑
0.72	131.0946	L-Leucine	1.90	5.10*E* − 03	2.47	↑
10.64	423.3348	Linoleyl carnitine	1.60	2.02*E* − 02	2.77	↑
0.70	174.1115	L-Arginine	1.51	2.91*E* − 02	2.01	↑
4.74	179.0582	Hippuric acid	1.36	4.99*E* − 02	0.35	↓
0.74	113.059	Creatinine	1.93	4.24*E* − 03	2.25	↑
5.93	148.0375	Citramalic acid	1.50	2.93*E* − 02	2.08	↑
7.38	736.7261	CE(24:0)	1.97	3.40*E* − 03	2.93	↑
0.71	161.1054	Carnitine	1.85	6.40*E* − 03	0.50	↓
12.16	455.3972	Arachidyl carnitine	1.86	6.23*E* − 03	1.89	↑
6.48	175.0635	3-Indoleacetic acid	2.06	2.11*E* − 03	2.04	↑
10.67	196.1462	2E,4E-Dodecadienoic acid	1.51	2.91*E* − 02	0.53	↓
0.72	168.0282	Uric acid	1.70	1.44*E* − 02	1.21	↑
7.64	455.1953	PS(6:0/6:0)	2.13	1.73*E* − 03	0.17	↓
11.89	549.3038	PS(20:2(11Z,14Z)/0:0)	1.65	1.79*E* − 02	2.34	↑
14.14	256.2395	Palmitic acid	2.00	3.55*E* − 03	0.72	↓
10.34	450.2617	PA(19:1(9Z)/0:0)	1.73	1.26*E* − 02	0.45	↓
1.00	190.0098	Oxalosuccinic acid	1.40	4.72*E* − 02	1.47	↑
14.30	282.2553	Oleic acid	1.83	8.11*E* − 03	0.70	↓
13.57	280.2398	Linoleic acid	1.85	7.25*E* − 03	0.71	↓
0.68	146.0696	L-Glutamine	1.56	2.61*E* − 02	0.59	↓
4.73	179.0576	Hippuric acid	1.49	3.39*E* − 02	0.30	↓

## Data Availability

The data (excel files) used to support the findings of this study are currently under embargo while the research findings are commercialized. Requests for data (12 months) after publication of this article will be considered by the corresponding author.
